# Structure and function of *Bs*164 β-mannosidase from *Bacteroides salyersiae* the founding member of glycoside hydrolase family GH164

**DOI:** 10.1074/jbc.RA119.011591

**Published:** 2019-12-22

**Authors:** Zachary Armstrong, Gideon J. Davies

**Affiliations:** Department of Chemistry, Structural Biology Laboratory, The University of York, York YO10 5DD, United Kingdom

**Keywords:** carbohydrate chemistry, enzyme catalysis, structural biology, glycoside hydrolase, glycosidase, carbohydrate processing, carbohydrate-active enzyme, conformational analysis, mannosidase, reaction mechanism

## Abstract

Recent work exploring protein sequence space has revealed a new glycoside hydrolase (GH) family (GH164) of putative mannosidases. GH164 genes are present in several commensal bacteria, implicating these genes in the degradation of dietary glycans. However, little is known about the structure, mechanism of action, and substrate specificity of these enzymes. Herein we report the biochemical characterization and crystal structures of the founding member of this family (*Bs*164) from the human gut symbiont *Bacteroides salyersiae.* Previous reports of this enzyme indicated that it has α-mannosidase activity, however, we conclusively show that it cleaves only β-mannose linkages. Using NMR spectroscopy, detailed enzyme kinetics of WT and mutant *Bs*164, and multiangle light scattering we found that it is a trimeric retaining β-mannosidase, that is susceptible to several known mannosidase inhibitors. X-ray crystallography revealed the structure of *Bs*164, the first known structure of a GH164, at 1.91 Å resolution. *Bs*164 is composed of three domains: a (β/α)_8_ barrel, a trimerization domain, and a β-sandwich domain, representing a previously unobserved structural-fold for β-mannosidases. Structures of *Bs*164 at 1.80–2.55 Å resolution in complex with the inhibitors noeuromycin, mannoimidazole, or 2,4-dinitrophenol 2-deoxy-2-fluoro-mannoside reveal the residues essential for specificity and catalysis including the catalytic nucleophile (Glu-297) and acid/base residue (Glu-160). These findings further our knowledge of the mechanisms commensal microbes use for nutrient acquisition.

## Introduction

Mannose is an essential component of protein human *N*-glycans (1), storage polymers, mannan and glucomannan in plants (2), and mannogen in *Leishmania* (3), and has even been observed to play a role in enzyme substrate recognition (4). Considering the roles of mannose containing polymers, it is no surprise that nature has developed a variety of enzymes to either modify the properties of these polymers or release their stored energy. The majority of enzymes that degrade mannose polymers are classified as glycoside hydrolases (GHs).^2^ The Carbohydrate Active Enzymes (CAZy) database (5) (http://www.cazy.org)^3^ categorizes all known GHs into >160 families, including several with known mannosidase activity. A major focus of the recent study has centered on how the enteric bacteria employ GHs to degrade a variety of polysaccharides including the mannose polymers present in the human gut (6–10), resulting in a better understanding of degradation mechanisms that underpin the nutrient acquisition by commensal bacteria.

Recent work by Helbert and co-workers (11) identified a new family of mannosidases: family GH164. This was accomplished through the combination of sequence space exploration, gene synthesis, and high-throughput activity assays. The founding member of GH164, which we shall herein refer to as *Bs*164, originates from the enteric bacterium *Bacteroides salyersiae* CL02T12C01 and was initially reported by the authors to have α-mannosidase activity. This family at present contains 17 genes, the majority of which belong to host-associated strains. However, due to the preliminary nature of this discovery very little is known about the structure, action mechanism, or substrate specificity of GH164 enzymes.

Here we present the detailed biochemical and structural analysis of *Bs*164. In contrast to initial reports, this enzyme has no α-mannosidase activity; instead, Bs164 cleaves β-mannosidic linkages in aryl β-mannosidase and mannooligosaccharides. X-ray crystal structures of *Bs*164 reveal a homotrimeric quaternary structure with each individual chain containing three domains. The catalytic domain of *Bs*164 consists of a (β/α)_8_ barrel with catalytic residues on β-strands 4 and 7 placing this family in clan GH-A. NMR analysis revealed a retaining mechanism and site-directed mutagenesis confirmed the assignment of the nucleophile and acid/base catalytic residues. Structures of inhibitor complexes show the conserved catalytic machinery involved in substrate recognition and catalysis and provide insight into the likely conformational itinerary for mannoside hydrolysis. Taken together, this work provides a thorough biochemical basis for the β-mannoside hydrolysis catalyzed by GH164 enzymes.

## Results and discussion

### Operonic context of GH164s

**Figure 1.**
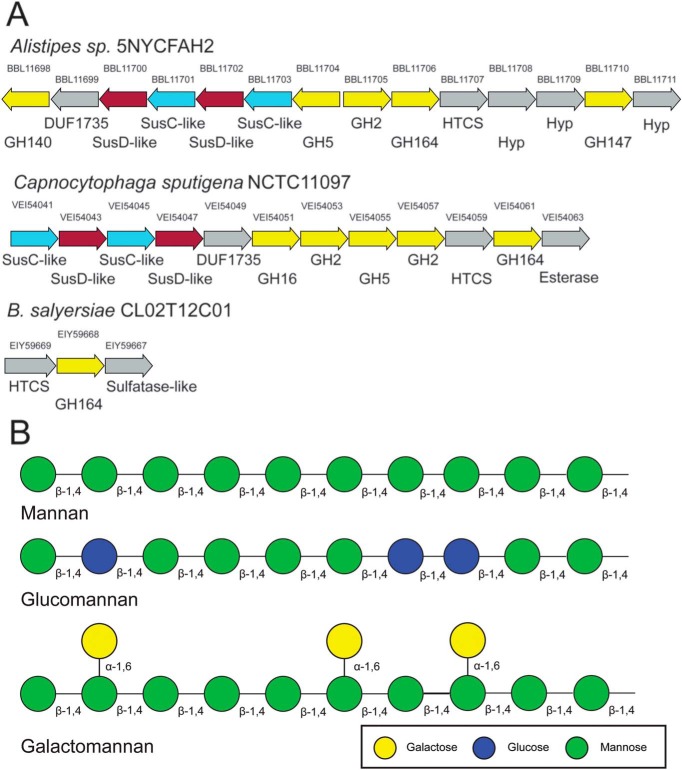
**Genomic context of GH164 enzymes and mannan structure.** *A,* genomic context of the GH164 genes present in the genomes of *B. salyersiae*, *Alistipes* sp., and *C. sputigena*. Locus tags are given *above the arrows* and predicted annotation is given *below*. Genes predicted to be GHs are highlighted in *yellow*, whereas the SusC-like and SusD-like proteins are shown in *blue* and *burgundy*, respectively. Genes are not shown to scale. Hybrid two-component systems are abbreviated *HCTS*, whereas hypothetical proteins are annotated *Hyp*. None of the GH5 or GH16 genes are currently annotated as belonging to specific subfamilies. *B,* mannans and glucomannans contain β-1,4-linked d-mannose residues in their structural backbone with glucomannans also containing backbone β-1,4-linked d-glucose residues. This backbone can be decorated with acetyl groups at the 2- and 3-positions or with α-linked galactosyl groups at the 6-position, forming galactoglucomannans (41).

The GH164 genes present in the CAZy database are confined to the Bacteroidetes (5), a phylum that is well-known for carbohydrate utilization operons (12). In addition to the GH164 from *B. saylersiae*, four genes originate from the *Alistipes* genus, commonly found in the human gut (13), 10 from the *Capnocytophaga,* typically found in the oralpharengyl tract (14), and one each belonging to the marine bacterium *Flammeovirga* and the lichen-associated *Mucilaginibacter*. Examination of the operonic context of the GH164 gives some clue to the polysaccharides, which they target (Fig. 1). The locus surrounding *Bs*164 is limited to two other genes, a hybrid two-component sensor and an arylsulfatase-like protein, however, the GH164 containing operons from *Alistipes sp.* 5NYCFAH2 and *Capnocytophaga sputigena* NCTC11097, see Fig. 1*A*, are much more elaborate. Both of these operons contain a SusC and SusD-like protein, the hallmark of polysaccharide utilization loci (12), as well as both endo- and exo-acting GH families. The presence of GH5, a family that contains endo-β-mannanases, and GH2, a family containing both β-galactosidases and β-mannosidases, and the absence of α-mannose targeting families suggests that these operons function to degrade dietary β-mannan, glucomannan, or galactomannan rather than *N*-glycans. The *B. salyersiae* genome also contains both GH5 and GH2 encoding genes suggesting that it may also target mannans, although with enzymes from separate loci.

### Bs164 is a retaining β-mannosidase

The *Bs*164 protein containing an N-terminal His_6_ tag was purified using immobilized metal affinity chromatography followed by size exclusion chromatography. The His_6_ tag was then removed using 3C protease, and the untagged protein was further purified using an additional size exclusion chromatography step. *Bs*164 eluted from the size exclusion earlier than would be expected for a 74.4-kDa protein. To determine whether *Bs*164 exists as a multimer in solution we subjected purified *Bs*164 to SEC-MALS (size exclusion chromatography with multiangle light scattering). A single peak was observed with a calculated molecular mass of 227 kDa, signifying that *Bs*164 forms trimers in solution.

**Figure 2.**
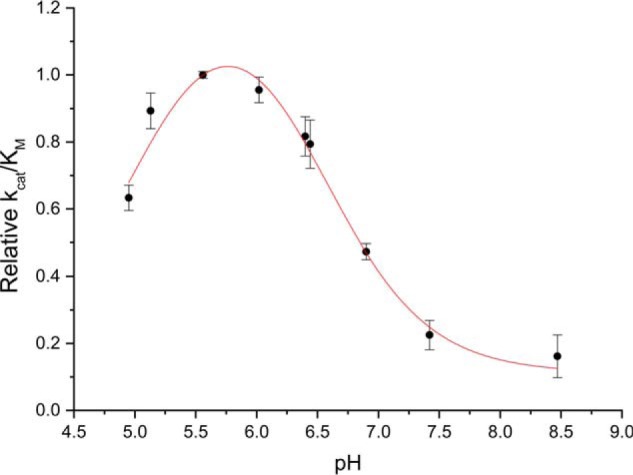
**pH profile of Bs164 activity.** Specificity constants for the hydrolysis of MU β-Man were determined between pH 5.0 and 8.5 using substrate depletion kinetics. MES buffer was used between 5.0 and 6.5 and HEPES buffer was used from pH 6.5 to 8.5.

To assess the substrate tolerance of *Bs*164 we assayed the purified enzyme with a variety of synthetic aryl glycosides and mannooligosaccharides (see “Experimental procedures” for a complete list). *Bs*164 shows no activity toward d-xylose, d- or l-arabinose, d-galactose, d-glucose, or l-fucose containing substrates. Furthermore, no activity was observed toward α-d-linked mannosides, contradicting the initial report of enzyme activity by Helbert *et al.* (11). *Bs*164 did, however, have significant activity against β-linked aryl mannosides with pH optimum 5.5 (Fig. 2). This pH was used in assays to characterize the specificity if this enzyme toward β-mannoside hydrolysis (Table 1). The specificity constant for *p*NP β-Man hydrolysis was ∼6 times that seen for methylumbelliferyl β-d-mannopyranoside (MU β-Man), which we attribute to *p*NP being a slightly better leaving group. This enzyme is also able to hydrolyze β-linked mannooligosaccharides and shows comparable specificity constants for the hydrolysis of mannobiose, mannotriose, and mannotetraose (Table 1) indicating that it contains a +1 subsite, but that binding in the +2 position does not greatly increase hydrolysis rates. The specificity constants for mannooligosaccharide hydrolysis were also 2–5 times lower than those seen for *p*NP β-Man, which we again attribute to the better leaving group ability of *p*NP.

**Table 1:** **Kinetic parameters of mannoside hydrolysis for *Bs*164 and mutants**

Enzyme	Substrate	*k*_cat_	*K*_M_	*k*_cat_/*K*_M_
		*s*^−*1*^	*mm*	*s*^−*1*^ *mm*^−*1*^
*Bs*164	*p*NP β-man	40 ± 3	3.6 ± 0.8	11 ± 3
*Bs*164	MU β-man	1.8 ± 0.05	1.05 ± 0.08	1.7 ± 0.1
*Bs*164	Mannobiose	–	–	2.2 ± 0.4
*Bs*164	Mannotriose	–	–	5.6 ± 0.7
*Bs*164	Mannotetraose	–	–	4.5 ± 0.7
*Bs*164_E297Q	*p*NP β-man	0.124 ± 0.003	0.87 ± 0.05	0.14 ± 0.01
*Bs*164_E160Q	*p*NP β-man	–	–	<10^−6^ *^a^*

*^a^* Estimated from limit of detection.

**Figure 3.**
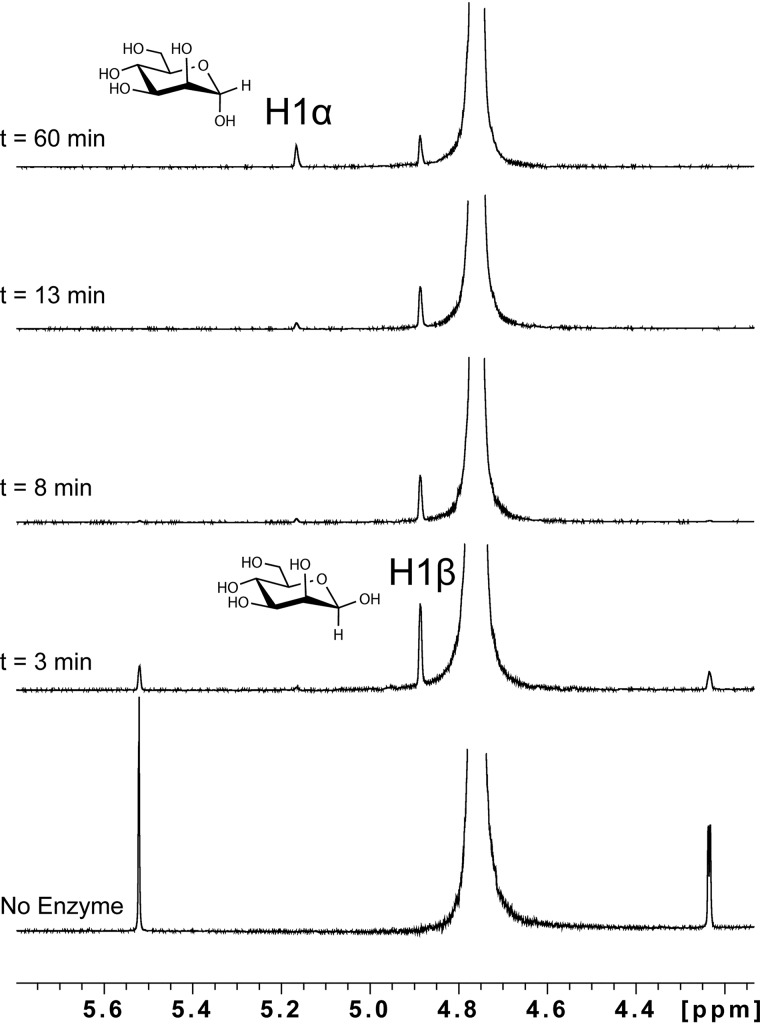
***Bs*164 acts with retention of stereochemistry.** pNP β-Man was incubated with *Bs*164 and the stereochemistry of the reaction was monitored by ^1^H NMR spectroscopy. Before enzyme addition there are no anomeric protons corresponding to free mannose. At *t* = 3 min, a single new peak corresponding to the H1β of mannose is observed at δ 4.88 ppm. Mutorotation of the anomer results in a decrease in the H1β signal and an increase of the H1α signal at δ 5.16 ppm over time.

We next sought to determine whether *Bs*164 employs a mechanism that results in either inversion or retention of the stereochemistry at the anomeric center. We used ^1^H NMR spectroscopy to monitor the enzyme-catalyzed hydrolysis of paranitrophenyl β-d-mannopyranoside (*p*NP β-Man) over the course of time (Fig. 3). This revealed that the β-anomer of mannose is produced immediately after the enzyme is added. The free mannose then undergoes mutorotation and the ratio between α- and β-anomers approaches equilibrium over time. This confirms that *Bs*164 is a retaining mechanism β-mannosidase and supports a mechanism that employs a nucleophile and an acid/base residue and transits through a glycosyl-enzyme intermediate.

### Crystal structure of Bs164 reveals a trimeric quaternary structure

**Figure 4.**
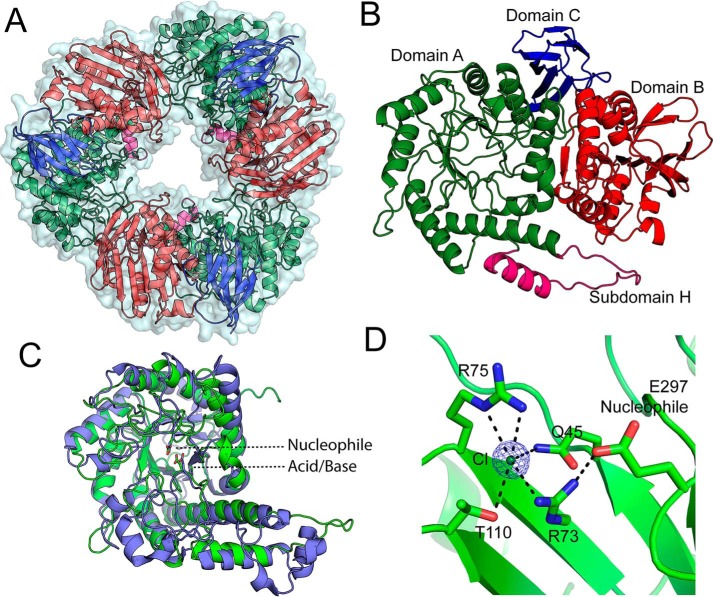
**Structure of *Bs*164.** The trimeric structure of *Bs*164 is shown in *panel A*. All three protomers are shown with a surface and each chain is displayed as a cartoon diagram colored by domain. *Panel B* shows the structure of one protomer. Domain A, which has a (β/α)_8_-fold, is shown in *green* with subdomain H shown in *magenta*, domain B, containing a mixed β-sheet, is shown in *red,* and the β-sandwich of domain C is shown in *blue*. Overlay of domain A of *Bs*164 and the catalytic domain of β-galactosidase from *Rahnella* sp. R3 (5E9A) is shown in *panel C. Bs*164 is in *green*, whereas 5E9A is colored *blue*. Both the catalytic nucleophile and acid/base residue of both structures are shown as *sticks*. The chlorine-binding site of *Bs*164 is shown in *D*, with *dashed lines* showing polar interactions closer than 3.4 Å apart. Electron density for the chloride is a σA-weighted 2*F_o_* − *F_c_* density contoured at 3 σ and rendered with the program PyMol. The nucleophile residue Glu-297 is also shown as it interacts with Arg-73.

We solved the structure of *Bs*164 using selenomethionine multiwavelength anomalous dispersion data to 2.3 Å and native protein data to 1.9 Å, see Table 2 for refinement statistics. The asymmetric unit of the P1 crystal form contains six *Bs*164 protomers arranged into two donut-shaped trimers, consistent with the trimeric form seen in solution (Fig. 4*A*). Each trimer-donut has an outer diameter of ∼100 Å and an internal diameter of between 30 and 35 Å. The individual *Bs*164 chains contain three clearly defined domains: a modified (β/α)_8_ barrel (residues 22–389), a domain containing a 7-membered mixed β-sheet sandwiched between α-helices (390–609), and a β-sheet domain (610–681) (Fig. 4*B*). This domain architecture is quite similar to that seen for family GH42 enzymes (15), but is previously unseen for β-mannosidases. Furthermore, analysis using the DALI server (16) indicates that the structure with highest similarity to *Bs*164 (RMSD = 2.4 Å, Z-score = 22.8, 100% coverage) is indeed a GH42 β-galactosidase from *Bacillus circulans* sp. a*lkalophilus* (PDB code 3TTY) also with trimeric quaternary structure (17).

**Table 2:** **Data collection and refinement statistics**

	SeMet-*Bs*164			“Apo”-*Bs*164 (6T5O)	2-F-Mannose (6T75)	Mannoimidazole (6T7G)	Noeruomycin (6T6G)
**Data collection**							
Space group	P1			P1	P1	P1	P1
Cell dimensions							
*a*, *b*, *c* (Å)	69.5, 104.5, 170.4			69.2, 103.9, 169.2	70.2, 104.9, 171.6	69.7, 104.7, 170.6	69.8, 105.1, 170.5
α, β, γ (°)	92.3, 97.4, 106.2,			92.5 97.3, 106.4	92.0, 97.7, 107.2	92.3, 97.3, 106.3	92.6, 97.2, 105.1
	Peak	Inflection	Remote				
Wavelength (Å)	0.9794	0.9797	0.9777	0.9795	0.9763	0.97623	0.9159
Resolution (Å)	67.13–2.30 (2.34–2.30)	67.13–2.30 (2.34–2.30)	67.13–2.30 (2.34–2.30)	66.76–1.91 (1.94–1.91)	99.90–2.54 (2.59–2.55)	168.66–1.79 (1.83–1.80)	101.13–2.06 (2.10–2.06)
*R*_merge_	0.070 (0.297)	0.074 (0.338)	0.086 (0.477)	0.062 (0.546)	0.113 (1.073)	0.044 (0.548)	0.115 (1.039)
*I/*σ*I*	15.3 (4.9)	15.3 (4.7)	14.5 (4.0)	9.4 (1.6)	6.1 (1.0)	6.5 (1.1)	5.2 (1.0)
Completeness (%)	99.9 (97.9)	99.9 (97.9)	99.9 (97.9)	95.6 (93.1)	98.7 (98.0)	100.0 (100.0)	98.3 (97.4)
Redundancy	7.0 (7.0)	7.0 (7.0)	7.0 (7.0)	3.2 (3.2)	3.6 (3.7)	2.0 (2.0)	3.5 (3.3)
CC_1/2_	0.99 (0.96)	0.99 (0.95)	0.99 (0.98)	0.99 (0.73)	0.99 (0.46)	0.99 (0.66)	0.98 (0.60)
**Refinement**							
Resolution (Å)				1.91	2.55	1.80	2.06
No. reflections				332,570	148,575	454,150	280,985
*R*_work_/*R*_free_				0.19/0.22	0.21/0.25	0.21/0.23	0.21/0.24
No. atoms							
Protein				31,506	31,388	31,597	31,482
Ligand/ion				175/6*^a^*	66/6	148/6	90/6
Water				1,902	159	1,666	1,029
*B*-factors (Å^2^)							
Protein				33	54	42	49
Ligand/ion				42/27	48/46	35/49	33/35
Water				34	40	43	37
R.M.S. deviations							
Bond lengths (Å)				0.008	0.009	0.008	0.01
Bond angles (°)				1.4	1.5	1.4	1.6

*^a^* Ligands in the uncomplexed structure are derived from ethylene glycol, tartrate/chloride.

**Figure 5.**
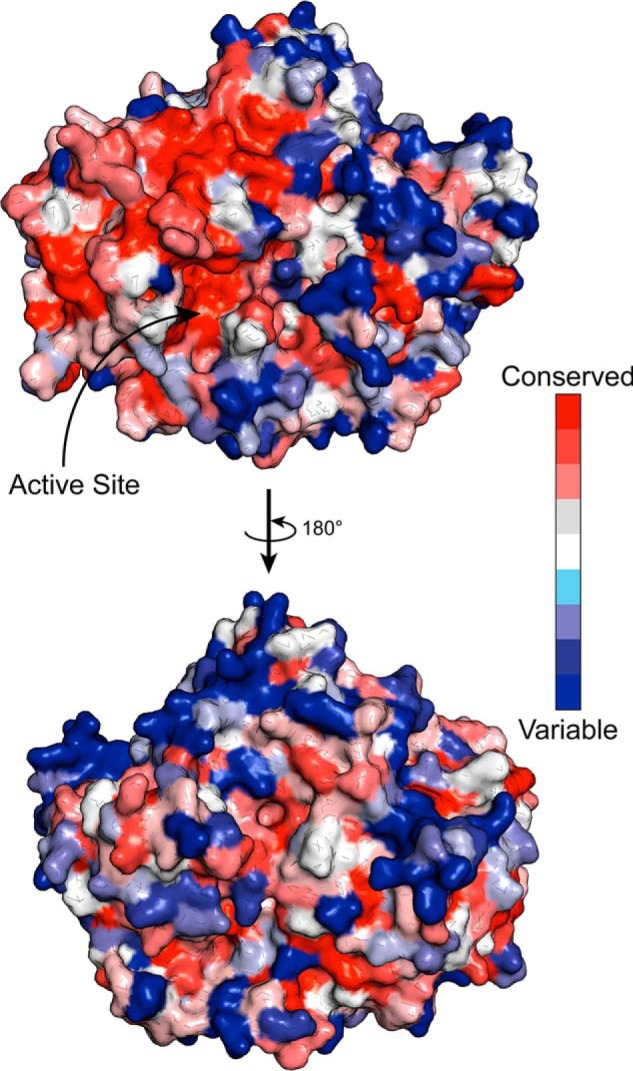
**Conservation of GH164 surface.** Surface representation of *Bs*164 colored by sequence conservation within the GH164 family. The figure was prepared using the CONSURF server (40) and generated in PyMol. The *top* representation is in the same orientation as described in the legend to Fig. 3*B*.

The (β/α)_8_-fold of domain A is found in the catalytic domain of many glycoside hydrolases and is a distinctive feature of clans GH-A, -D, -K, and -R. Domain A also contains a helix-containing subdomain, atypical of (β/α)_8_-folds but also observed in the GH42 family (15), which is present between the 4th β-strand and the next α-helix. This domain projects along the side of the domain B and interacts with domain A of a neighboring chain (Fig. 4*B*). We speculate that the presence of this domain helps, in part, to stabilize the trimeric structure. Domain A shows highest similarity, by means of a Dali search (16), to the catalytic domain from the GH42 β-galactosidase from *Rahnella* sp. (PDB ID code 5E9A) with RMSD of 3.2 Å and complete query coverage (18). Overlay of these two structures revealed that the nucleophile (Glu-314 in PDB 5E9A) and acid/base residue (Glu-157 in PDB 5E9A) are conserved in *Bs*164 and the putative catalytic nucleophile and acid/base are Glu-297 and Glu-160, respectively. Comparison of amino acid conservation within the GH164 family also shows that this active site is completely conserved across the family (Fig. 5). The positioning of the catalytic residues on strands 4 (acid/base) and 7 (nucleophile) indicate that the GH164 belongs to clan GH-A glycoside hydrolases, which contains in addition to GH42, several other families whose members are active on β-mannose linkages, namely: GH2, GH5, GH26, and GH113.

The active site of *Bs*164 is a shallow pocket that has a much more open + binding subsites than is seen in the exo-mannosidases *Bt*Man2A (9) and *Cm*Man5A (19). Both *Bt*Man2A and *Cm*Man5A contain tryptophan residues, Trp-470, for *Bt*Man2A and Trp-135 for *Cm*Man5A, in the positive subsites, constraining the active site, and creating a more tunnel-like environment, whereas *Bs*164 lacks an aromatic residue in a similar position. Although the positive subsites of *Bs*164 are more open, there are some interactions that are consistent with other mannosidases. The residue His-264 in particular most resembles a positive subsite interaction residue seen in other mannosidases. His-264 is in the same position as both Trp-289 in *Cm*Man5A and Trp-519 in *Bt*Man2A, which are thought to form a part of the +2 subsite in these proteins. The complete conservation of His-264 across the GH164 family, and its positioning suggests that this residue forms part of the +2 subsite in *Bs*164.

In addition to the active site, domain A also contains a chloride-binding site. The chloride ion coordinates Gln-45, Thr-110, and two arginine residues (Arg-73 and Arg-75) in a penta-coordinate, distorted square pyramidal geometry (see Fig. 4*D*). The chloride present is observed in all 6 chains in the crystal structure and is observed in all of the inhibitor complexes described below. This chloride is present at a site adjacent to the active site and one of the coordinating arginines, Arg-73, and forms a direct interaction with the catalytic nucleophile. Additionally, Arg-75 projects into the active site and the two η-nitrogens form hydrogen bonds with the 2- and 3-hydroxyls of the sugar residue bound at the −1 subsite, as will be described below.

Domain B bears a striking resemblance to the trimerization domain of GH42 enzymes. In GH42 structures (15, 18, 20) there are a number of interactions between the trimerization domain and the catalytic domain that support trimeric quaternary structure. Likewise, domain B of *Bs*164 interacts over a surface area of 1380 Å^2^ per protomer that includes seven residues involved in direct hydrogen bonds and two salt bridges between chains. The corresponding interface in domain A consists of the loops connecting the last two pairs of β-sheets and α-helices. This trimerization also likely has a role in catalysis as the interaction of residues Ser-342–Ala-345 and Ala-347 in domain A with the domain B of the neighboring protomer appears to help position the active site residues Arg-341 and Glu-346 that interact directly with the substrate, as shown in the inhibitor-bound structures described in the next section.

Unlike the first two domains, function of the C-terminal β-sandwich domain is much more difficult to infer from the structure. This domain is also present in GH42 enzymes (15, 18, 20), however, the role of this domain within GH42s is unknown.

### Active center structure and catalysis

**Figure 6.**
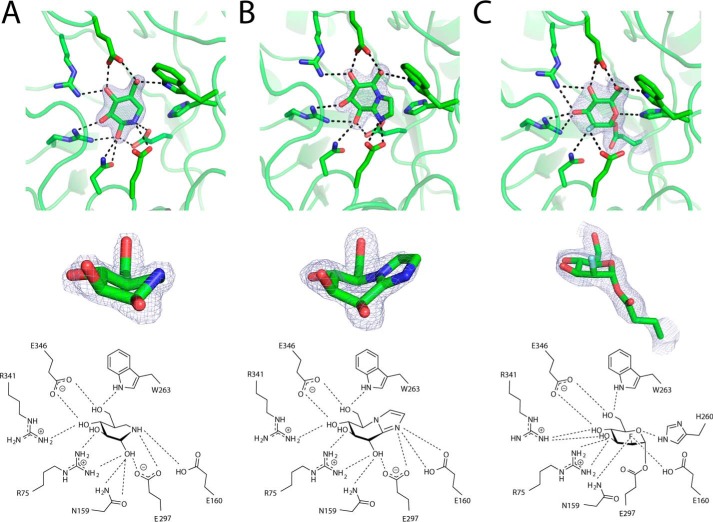
**Complexes of *Bs*164 with inhibitors.** *A,* complex with noeuromycin; *B,* complex with mannoimidazole; and *C,* a complex with covalently bound 2-deoxy-2-fluoromannose. The *top panel* shows the inhibitor within the active site with polar interactions within 3.2 Å shown as *black dashed lines*. The *middle panel* shows the inhibitor with the surrounding electron density, and the *bottom panel* shows a cartoon schematic of active site interactions. All electron density meshes are σA-weighted 2*F_o_* − *F_c_* densities contoured at 1.5 σ and rendered with the program PyMol. Trp-336, which sits below the inhibitors, has been omitted for clarity.

To gain further insight into the mechanism and substrate recognition and confirm the assignment of the catalytic residues we produced structures of *Bs*164 in complex with 2,4-dinitrophenyl 2-deoxy-2-fluoro-mannoside (DNP-2FM), noeuromycin, and mannoimidazole through soaks of *Bs*164 crystals. Mannoimidazole and noeuromycin act as competitive inhibitors of *Bs*164 with inhibition constants (*K_i_*) of 470 ± 60 μm and 340 ± 40 nm, respectively. The inhibition constant for noeuromycin is well in line with previous reports of β-mannosidase inhibition (21, 22), however, inhibition by mannoimidazole notably is much worse than is seen for other β-mannosidases such as BtMan2A (*K_i_* = 1.4 μm) (21). The fluorosugar DNP-2FM is a mechanism-based inhibitor that forms a glycosyl-enzyme intermediate that is turned over at much slower rates than the natural sugar, thereby enabling the identification of the catalytic nucleophile (23). All three of these inhibitors are present in the *Bs*164 active site −1 subsite (Fig. 6).

The mannosidase inhibitors are recognized in the −1 subsite by eight polar amino acids, which are within hydrogen bonding distance to the substrate (Fig. 6). All eight of these polar contacts are completely conserved across all GH164s currently within the CAZy database, and form similar interactions with each of the inhibitors. The O6 sits pointing toward the β-face between the carbonyl of Glu-346 and the indole nitrogen of Trp-263. Glu-346 is also positioned to hydrogen bond O4 of the inhibitors, the Glu-O4-O6 motif is seen in a number of mannosidase structures including those for GH1 (PDB ID 4RE2) (24), GH2 (PDB ID 2VMF) (21), and GH5 (PDB ID 1UZ4) (22). The O4 of all inhibitors hydrogen bonds with Arg-341, is part of a loop that interacts with the trimerization domain of the neighboring protomer. Another arginine, Arg-75 (which is bound to chlorine) hydrogen bonds to O3 of all inhibitors and the O2 of mannoimidazole and noeuromycin. Arg-75 lies below the sugar plane and likely provides impetus for the O2 axial to equatorial migration, the presence of an arginine bridging the O2 and O3 hydroxyls is also seen in the GH113 β-mannanase *Aa*ManA (25). The O2 hydroxyl also hydrogen bonds to Asn-159 in all three inhibitor structures and the nucleophile (Glu-297) in mannoimidazole and noeuromycin structures. The hydrophobic element on the α-face of the sugar is provided by Trp-336, well within the range to form CH-π interactions, a motif that is often seen in protein–β-mannose interactions (26).

The structure of bound 2FM clearly shows a covalent intermediate attached to Glu-297, this observation along with kinetic analysis of an E297Q variant conclusively identifies Glu-297 as the active site nucleophile. The acid/base residue, Glu-160, is positioned to perform *anti*-protonation of the leaving group, typical of clan GH-A glycoside hydrolases (27). This residue forms hydrogen-bonding interactions with both the endocyclic nitrogen in noeuromycin and imidazole nitrogen in mannoimidazole, an interaction that is also seen in GH26 and GH113 β-mannanases (25) and GH2 β-mannosidases (21). Further evidence, in the form of near complete loss of activity by the E160Q variant (see Table 1) confirms the assignment of Glu-160 as the acid/base residue.

The inhibitor-bound structures of *Bs*164 also give insight into the conformational itinerary of β-mannoside hydrolysis. Most β-mannosidases and β-mannanases, including families GH2, GH5, GH26, GH113, and GH130 have been observed to transit through a ^1^S_5_ → B_2,5_^‡^ → ^O^S_2_ glycosylation itinerary (28), the outlier being the inverting enzyme GH134 that instead proceeds through ^1^C_4_ → ^3^H_4_^‡^ → ^3^S_1_ (see Ref. 29). The structure of the mannoimidazole, an inhibitor that faithfully reports on the transition state of mannosidases (21, 25, 28, 30), is clearly in a B_2,5_ conformation within the *Bs*164 active site (Fig. 6*B*) indicating that the conformational itinerary is centered, as for most β-mannosidases, around a B_2,5_ transition state. The structure of the noeuromycin complex, in which a ^1^S_5_ conformation is observed (Fig. 6*A*), lends further support to this itinerary. Observations from other β-mannosidases (28, 31) suggest we should expect the glycosyl-enzyme intermediate to adopt a ^O^S_2_ conformation, however, in the complex with 2FM the ring is in a ^4^C_1_/E_5_ conformation (ϕ = 295°, θ = 37°) with the 2F positioned axially, instead of equatorially as would be expected in a ^O^S_2_ conformation. The comparatively low resolution of the 2FM structure (2.55 Å) makes it difficult to equivocally confirm the conformation of the glycosyl-enzyme intermediate that appears to refine as ^4^C_1_/E_5_. We endeavor to further explore the energetic consequences of this ^4^C_1_/E_5_ conformation and whether this structure is representative of the true glycosyl-enzyme intermediate or an energetic relaxation of this intermediate to a position that is not along the conformational itinerary.

## Conclusions

The capacity of commensal bacterial to digest dietary carbohydrates relies on a broad range of enzymes, tailored to their specific target. Examination of *Bs*164, the prototypical GH164, adds to our growing understanding of the mechanisms underpinning enteric carbohydrate degradation. Our interrogation of this enzyme has shown that it exists as a donut-shaped homotrimer, a new domain architecture and quaternary structure for β-mannosidases, and employs two conserved glutamic acid residues in a retaining mechanism. The structural analysis of *Bs*164 in the presence of inhibitors revealed a host of active-site interactions and suggested a boat-like transition state. Further investigation of how the genes co-localized within GH164 containing operons, promises to reveal how these loci function in concert to enable degradation of mannose containing polymers within the human microbiome.

## Experimental procedures

### Substrates and inhibitors

Paranitrophenyl β-d-mannopyranoside and methylumbelliferyl β-d-mannopyranoside were purchased from Sigma-Aldrich. Mannobiose, mannotriose, and mannotetraose were purchased from Megazyme (Bray, Ireland). Mannoimidazole and noeuromycin were kind gifts from Professor Spencer Williams and Professor Robert V. Stick, respectively. 2,4-Dinitrophenyl 2-deoxy-2-fluoro-β-d-mannopyranoside was a kind gift from Professor Stephen G. Withers (University of British Columbia).

### Gene expression and protein purification

The gene encoding *Bs*164 (GenBank^TM^ accession EIY59668.1) was predicted to contain a signal peptide with a cleavage site between amino acids 21 and 22 by signalP 5.0 (32). A codon optimized version of this gene with a His_6_ tag in place of the signal peptide (MGSSHHHHHHSSGLEVLFQGPA) was synthesized by and cloned into a pET-28 vector by GenScript (Leiden, Netherlands). Plasmid was transformed into chemically competent BL21(*DE3*) gold cells (Agilent) and plated on LB agar containing 50 μg/ml of kanamycin. A single colony was used to inoculate 5 ml of LB media containing 50 μg/ml of kanamycin. After overnight growth at 37 °C with shaking at 180 rpm this starter culture was used to inoculate NZYTech autoinduction media (NZYTech) containing 50 μg/ml of kanamycin. Expression cultures were grown at 37 °C with shaking at 250 rpm for 6 h, the temperature was then decreased to 20 °C and cultures were incubated for an additional 22 h. Expression cultures were harvested by centrifugation (5,000 × *g*, 30 min, 4 °C) and cell pellets were stored at −80 °C until purification.

To purify the protein cell pellets were resuspended in 120 ml of Buffer A (50 mm HEPES, 30 mm imidazole, 200 mm NaCl, pH 7.4) with additional protease inhibitor (4-(2-aminoethyl)benzenesulfonyl fluoride, 0.1 mm) lysozyme, and DNase. Resuspended cells were then lysed by passage through a cell-disruptor homogenizer at 25 kpsi. Lysed cells were centrifuged (18,000 × *g*, 30 min, 4 °C) and the supernatant was decanted from the cell debris. Clarified supernatant was loaded directly onto a 5-ml His tag Excel column (GE Healthcare). Bound protein was washed with 7.5 column volumes of buffer A then eluted with a linear gradient of 0–100% buffer B (50 mm HEPES, pH 7.4, 1 m imidazole, 200 mm NaCl) over 20 column volumes. Eluted protein was concentrated with a 30-kDa cut-off Amicon centrifugal filter unit and further purified by gel filtration (HiLoad 16/600 Superdex 200 pg; GE Healthcare) in buffer C (50 mm HEPES, 200 mm NaCl, pH 7.4). The purity of eluted protein was analyzed by SDS-PAGE and the peak fractions were pooled and concentrated with a 30-kDa cutoff Amicon centrifugal filter unit. Concentrated protein was washed with buffer D (20 mm HEPES, pH 7.4) then diluted to 30 mg/ml with buffer D and flash frozen with liquid nitrogen until use. Protein concentrations were determined spectrophotometrically using a calculated *A*_280_ extinction coefficient of 128,480 m^−1^ cm^−1^. For biochemical studies, the N-terminal His_6_ tag was cleaved from *Bs*164 through overnight incubation with a 1:100 ratio (by mass) of 3C-protease. Cleaved *Bs*164 was then purified by gel filtration and concentrated as detailed above. The average yield of purified protein was ∼30 mg/liter of culture.

Selenomethionine-labeled protein was expressed using the same bacterial strain as for the WT protein. This strain was used to inoculate 40 ml of LB media containing 50 μg/ml of kanamycin, which was grown overnight at 37 °C. This culture was then centrifuged (3,000 × *g*, 10 min, 4 °C) and the cell pellet was resuspended in 40 ml of M9 media containing 50 μg/ml of kanamycin. The resuspended cells (1 ml) were then used to inoculate a 1-liter culture of M9 media containing 50 μg/ml of kanamycin. This culture was incubated at 37 °C with shaking at 250 rpm until mid-log phase (A_600_ = 0.5). At this point lysine (100 mg/liter), phenylalanine (100 mg/liter), threonine (100 mg/liter), isoleucine (50 mg/liter), leucine (50 mg/liter), valine (50 mg/liter), and selenomethionine (50 mg/liter) were added to the culture, which was incubated for another 15 min at 37 °C. The incubation temperature was then lowered to 20 °C and isopropyl 1-thio-β-d-galactopyranoside was added to a final concentration of 0.5 mm. After incubation for 18 h the cultures were harvested as for the unlabeled protein. The selenomethionine protein was purified as for the unlabeled protein except for the addition of 5 mm DTT to buffers A, B, and C and the addition of 0.5 mm Tris(2-carboxyethyl)phosphine to buffer D.

Mutagenesis was performed using a modified QuikChange^TM^ (Agilent) protocol. For each mutant generated, PCR was first performed for 12 cycles with one of the sense or antisense primers (E288Q_F: GTG GCT GAT GAC CCA ACT GCA AGG TG; E288Q_R: CAC CTT GCA GTT GGG TCA TCA GCC AC; E151Q_F: GTG CTG ATT AAC CAG CCG GGT ACC C; E151Q_R: GGG TAC CCG GCT GGT TAA TCA GCA C); these two reactions were subsequently pooled, and an additional 18 cycles of PCR were performed. PCR products were digested with DpnI (New England Biolabs) and transformed into chemically competent BL21(*DE3*) gold cells. Purified plasmids were sequenced to confirm the correct mutation prior to expression. Both the nucleophile and acid base variants were purified as for the WT protein.

### Activity assays

All *Bs*164 activity assays, unless otherwise stated, were performed at 25 °C in a buffer containing 80 mm MES (pH 5.5), and 160 mm NaCl, and were initiated by the addition of the appropriate amount of enzyme. Purified *Bs*164 was initially tested for activity on the following aryl glycosides: *p*NP α-l-arabinofuranoside, *p*NP α-l-arabinopyranoside, *p*NP α-l-fucopyranoside, *p*NP β-d-galactopyranoside, *p*NP α-d-glucopyranoside, *p*NP β-d-glucopyranoside, *p*NP α-d-mannopyranoside, *p*NP β-d-mannopyranoside, and *p*NP β-d-xylopyranoside. Purified enzyme was added (final concentration of 1 μm) to a solution of 1 mm substrate. These assays were incubated at 25 °C and stopped after 6 h with a 1:1 ratio of 1 m Na_2_CO_3_ (pH 11.2) and absorbance at 400 nm was measured with a CLARIOstar Plus plate reader (BMG Labtech).

The optimal pH for activity was determined with MU β-Man using substrate depletion kinetics. Purified enzyme was added to solutions containing 50 μm MU β-Man, 160 mm NaCl, and either MES (pH 5–6.5) or HEPES (pH 6.5–8.5). Reactions were limited to this pH range as enzyme denaturation was observed above pH 8.5 and below pH 5. Fluorescence was monitored continuously (λ_ex_ = 365 nm, λ_em_ = 450 nm) and depletion curves were fit to the equation: *A* = *A_f_*(1-*e*^−^*^kt^*), where *k* = [*E*] · *k*_cat_/*K*_M_, to give the specificity constant at each pH.

Kinetic parameters were determined for *p*NP β-Man and MU β-Man using substrate concentrations covering the range of 1/5 to 5 times the eventual *K*_M_ determined. Assays were performed in 96-well-plates and each plate included either *p*NP or MU standards in an identical buffer system for concentration calibration. MU β-Man hydrolysis was monitored continuously (λ_ex_ = 365 nm and λ_em_ = 450 nm), whereas *p*NP β-man hydrolysis was measured after stopping the assay with a 1:1 ratio of 1 m Na_2_CO_3_ (pH 11.2).

The specificity constant for the hydrolysis of mannobiose, mannotriose, and mannotetraose was determined using an assay for reducing sugars employing bicinchonic acid. Reactions containing between 0.1 and 4 mm mannobiose were incubated at 25 °C and aliquots were removed over a period of 30 min. These aliquots were added to an equal volume of freshly prepared bicinchonic acid reagent containing: 400 mm sodium carbonate (pH 11.2), 2.5 mm bicinchonic acid, 1.25 mm CuSO_4_, and 2.5 mm l-serine. This mixture was heated to 80 °C for 10 min, and then cooled. The absorbance at 563 nm was measured with a CLARIOstar Plus plate reader (BMG Labtech) and compared with a standard curve of mannose to calculate the concentration of reducing sugars. Data were fit to linear equation to give the specificity constant.

To determine inhibition constant (*K_i_*) values Michaelis-Menten parameters were determined for *Bs*164 hydrolysis of pNP β-Man in reactions containing either mannoimidazole or noeuromycin. Assays were started with the addition of enzyme to the assay mix. Parameters were determined for at least 3 different inhibitor concentrations. Inhibition constants were calculated according to a competitive inhibition model for both inhibitors. All kinetic parameters were fit with the software program Origin 2019.

### SEC-MALS

Experiments were conducted on a system comprising a Wyatt HELEOS-II multiangle light scattering detector and a Wyatt rEX refractive index detector linked to a Shimadzu HPLC system (SPD-20A UV detector, LC20-AD isocratic pump system, DGU-20A3 degasser and SIL-20A autosampler). Work was conducted at room temperature (20 ± 2 °C). Sample injection volume was 100 μl at a protein concentration of 5 mg/ml. The samples were separated on a Superdex S200 10/300 (GE Healthcare) using 80 mm MES (pH 5.5), 200 mm NaCl as buffer. Shimadzu LC Solutions software was used to control the HPLC and Astra V software for the HELEOS-II and rEX detectors. Data were analyzed using the Astra V software. Molecular weights were estimated using the Zimm fit method (33) with 1 degree. A value of 0.19 was used for protein refractive index increment (dn/dc).

### NMR

Prior to NMR experiments buffer (80 mm MES, pH 5.5, and 160 mm NaCl), *p*NP β-mannoside and *Bs*164 were lyophilized. Buffer and enzyme were resuspended in D_2_O, whereas *p*NP β-mannoside was resuspended in 10% *d*_6_-DMSO in D_2_O. The final reaction mixture contained 4.5 mm *p*NP β-Man, 9 mm MES (pH 5.5), 145 mm NaCl, and 0.9% (v/v) *d*_6_-DMSO. NMR spectra were collected before the addition of enzyme and every 5 min after the addition of *Bs*164 to a final concentration of 120 μg/ml. NMR data were collected on a Bruker 700 MHz Avance Neo spectrometer equipped with a 5-mm triple resonance cryoprobe. 1-D proton spectra were recorded with a 30-degree excitation pulse, an interscan delay of 10 s, and a total acquisition time of 3 min. Sample temperature was maintained at 298 K, and referencing was relative to disuccinimidyl suberate.

### Crystallization and structure determination

His_6_-*Bs*164 at 30 mg/ml was tested against a range of commercial crystallization screens. Large split crystals were found in 100 mm ammonium tartrate dibasic (pH 7.0), 12% (w/v) PEG 3,350, a condition that was used for further optimization. Crystals formed in 0.1 m ammonium tartrate dibasic at a pH range from pH 5.5 to 7.0. The optimized crystals were grown in maxi 48-well-plates using the sitting-drop vapor-diffusion method at 20 °C with 100 mm ammonium tartrate dibasic (pH 7.0), 13% (w/v) PEG 3,350 with a protein:well solution ratio of 500:500 nl. Crystals were soaked in well solution containing 25% (v/v) ethylene glycol before flash cooling in liquid nitrogen. Inhibitor complexes were obtained by soaking crystals in well solution containing 10 mm of the inhibitor for 3–4 h before flash cooling in liquid nitrogen. For the structures with DNP-2FM solid substrate was added to the drop containing crystals, which were then soaked overnight. The overnight soaking with crystals of DNP-2FM gave inhibitor complexes, but did appear to reduce diffraction quality. Refinement statistics are given in Table 2.

*Bs*164 selenomethionine derivative, mannoimidazole, noeuromycin, and DNP-2FM diffraction data were collected at Diamond beamlines, and data were processed with the CCP4i2 suite (34). The unliganded structure of *Bs*164 was solved by MAD phasing of a selenomethionine derivative using 6 datasets: two at the peak wavelength (0.9794Å) using 0.1° oscillation and χ angle of 25° or 0°, for 360°; two at a remote wavelength (0.9777 Å) using 0.1° oscillation and χ angle of 25° or 0°, for 360°; and two at the inflection (0.9797 Å) using 0.1° oscillation and χ angle of 25° or 0°, for 360°. Crank2 (35) was used for phasing and initial model building. Cycles of maximum-likelihood refinement using REFMAC5 (36) were interspersed with manual corrections of the models using COOT (37). Complexed structures of *Bs*164 were solved by molecular replacement using the unliganded coordinates as the search model in Phaser (38). Structural figures were drawn with PyMol (DeLano Scientific LLC.).

### Sequence alignment

All 17 GH164 sequences currently available in the CAZy database (5) (accessed 01–12-2019) were aligned using the COBALT multiple sequence alignment tool (39) and amino acid conservation was determined using the ConSurf server (40) and visualized in PyMol.

### Data availability

The atomic coordinates and structure factors have been deposited in the Protein Data Bank using accession codes 6T5O, 6T75, 6T6G, and 6T7G.

## Author contributions

Z. A. data curation; Z. A. formal analysis; Z. A. and G. J. D. validation; Z. A. investigation; Z. A. visualization; Z. A. methodology; Z. A. writing-original draft; G. J. D. conceptualization; G. J. D. resources; G. J. D. supervision; G. J. D. funding acquisition; G. J. D. project administration; G. J. D. writing-review and editing.

## Footnotes

3 Please note that the JBC is not responsible for the long-term archiving and maintenance of this site or any other third party hosted site.

## Footnotes

2 The abbreviations used are:

GH: glycoside hydrolase
CAZy: carbohydrate active enzyme database
DNP-2FM: 2,4-dinitrophenyl 2-deoxy-2-fluoro-β-D-mannopyranoside
MU: 4-methylumbelliferyl
MU β-Man: 4-methylumbelliferyl β-d-mannopyranoside
PDB: Protein Data Bank
*p*NP: paranitrophenyl
*p*NP β-Man: paranitrophenyl β-d-mannopyranoside
RMSD: root mean square deviation.
